# Traditional behavioural practices, the exchange of saliva and HHV-8 transmission in sub-Saharan African populations

**DOI:** 10.1038/sj.bjc.6601390

**Published:** 2003-11-11

**Authors:** J M Wojcicki

**Affiliations:** 1 Center for AIDS Prevention Studies (CAPS), University of California, San Francisco (UCSF), 74 New Montgomery Street, Ste 600, Box 0886, San Francisco, CA 94105, USA

**Sir**,

With the discovery of human herpesvirus 8 (HHV-8) or Kaposi sarcoma (KS)-associated herpesvirus as the necessary but not sufficient causal agent in the development of Kaposi's sarcoma, there has been much interest in elucidating risk factors for transmission. The age-dependent increase of HHV-8 seroprevalence in childhood in endemic areas such as sub-Saharan Africa suggests some type of nonsexual horizontal transmission. As saliva is the bodily fluid that most often harbours HHV-8 and at greatest concentrations, it is likely that behaviours involving the exchange of saliva may facilitate transmission (Pauk *et al*, 2000). However, the exact behaviours that increase risk for HHV-8 transmission have not been delineated.

In order to examine potential behavioural risk factors associated with the exchange of saliva, I examined the Human Relations Area Files (HRAF), an on-line database organised by Yale University of over 350 000 pages of information on cultural and social life of different peoples (HRAF, 1997). I conducted searches using the terms, ‘saliva’, ‘spit’ and ‘spitting’ within this database so as to examine behavioural practices associated with the exchange of saliva in sub-Saharan African cultures. The results of this search include material from 12 ethnic groups (Bemba (Zambia), Nuer (Sudan), Ovimbundu (Angola), Igbo (Nigeria), Somali (Somalia), Wolof (the Gambia Senegal), Bena (Tanzania), Masai (Kenya/Tanzania), Lozi (Zambia), Banyoro (Uganda), Ashanti (Ghana), Azande (Sudan)) using 19 different source materials. As many of these sources are ‘classic’ ethnographies on different cultural practices, which were written by anthropologists, travellers and missionaries in the 20th century (from early 20th century to mid-20th century), it is necessary that future cross-sectional and longitudinal studies investigate and evaluate: (1) the extent to which some of these behaviours continue to be practiced among the ethnic groups discussed in this review and (2) whether they increase risk for infection with HHV-8 in children and adults.

Three types of behavioural practices associated with saliva exchange in sub-Saharan Africa emerged from this search: (1) the use of saliva in healing and medical practices, (2) the use of saliva in initiation or ritual practices and (3) the use of saliva in feeding practices.

Among some ethnic groups in the materials reviewed, it has been observed that part of the healing and treatment process involves using saliva to treat different ailments. Saliva alone or saliva combined with local herbs is believed to have medicinal properties. Among the Somali, sores that do not heal and have pus are treated with saliva (Cerulli, 1964, p. 9). It has also been observed among the Somali that the treatment for snakebites or scorpion stings involved mixing saliva with butter and applying the mixture to open wounds (Puccioni, 1936, p. 167). Other reports suggest that saliva is used in the treatment of all forms of disease among the Somali (Helander, 1988, p 111). Similarly, among the Azande of Sudan, saliva is employed as a first-aid application to wounds and abrasions (Anderson, 1911, p. 250a). Among some ethnic groups, herbs are chewed and the mixture of saliva and herbs is placed on the wound. In Tanzania, among the Bena, for the treatment of a boil, the traditional healer chews traditional herbs (*mhefefa* and *munepa*) and subsequently uses the chewed and softened herbs to treat wounds (Culwick, 1935, p. 395). Among the Masai of East Africa, the juice of the plant *ol giloriti* (*Acacia abyssinica*) is chewed and spit onto the wound by a healer as an astringent (Merker, 1910, p. 247). Similarly, so as to combat swelling, the chewed plant *ol agaram* (*Crossandra nilotica*) is placed on the swollen area (Merker, 1910, p 249) and a mother will place the chewed paste of plants called *ofe*, *n gilo* and *no orekum* (*Spilanthes acmella* L.) on the nostrils of a small child whose nose has been bitten by flies (Merker, 1910, p 251). A sucking cure has also been reported among the Azande of Sudan where the patient's body is sucked vigorously by the traditional healer (Rattray, 1923, pp 248–250). Also among the Igbo of Nigeria, it is observed that a traditional healer (*dibia*) will suck the arms, head or abdomen of a new-born baby in order to remove worms and will subsequently symbolically spit the worms out of his mouth (Meek, 1970, p 85). It is not clear if sucking is a cure that is widely practiced among different African groups or in different areas and what its relationship to HHV-8 infection might be.

A number of African studies report the use of spitting as a form of treatment with the traditional healer routinely spitting on the patient (Hollis, 1905; Helander, 1988). This search of the HRAF found spitting as a medicinal cure reported among the Masai (Hollis, 1905, p 315) and Somali of East Africa (Helander, 1988). As part of a blessing ceremony conducted by a man of religion (*wadaad*) or a layman among the Somali, it is required that the ill person be spit on so as to transfer the blessing (Helander, 1988, p 111).

This review found that in some African communities, certain birth rituals involve the use of saliva. Among the Wolof of West Africa, when a baby is born, it is reported that an elderly woman will visit the mother and infant and subsequently give the infant a blessing by spitting on its face and kissing it. Similarly, male elders will also covey blessings on the child by spitting into its ear and rubbing saliva over the infant's head. Among the Wolof, saliva has the power to retain words such as blessings. It is stated that, ‘Like honey in water, speech, good or bad, dissolves in saliva which retains part of its power’ (Gamble, 1991, p 152). Also, among the Igbo of Nigeria, there are also birth rituals associated with the use of saliva. After a child is born and begins crying, s/he is carried to the ancestral house where any kinsman who is known as an orator will chew grains of alligator pepper, spit the mixture onto his finger and insert it into the infant's mouth beneath the tongue. She/he will then rub the mixture on the genioglossi and exhort the child to become an orator (Henderson, 1966, p 18). Similarly, among the Ashanti of Ghana, after the birth of a child and at her/his naming ceremony, the child's grandfather will spit into the child's mouth to strengthen her/his spirit (Rattray, 1923, p 54).

Saliva may also be used in some of the activities or rituals of daily life. Contexts in which saliva may be used include spitting as a form of greeting. Among the Nuer of Southern Sudan, it is noted that men spit on the heads of their children on returning from trips and young girls who have not seen each other for some time spit on each other as a form of greeting (Huffman, 1931, p 87). Spitting as a greeting may also take place to reinforce social hierarchies. Among the Lozi of Zambia, senior chiefs or royal persons displayed favour and praise by placing saliva on the commoner's tongue (Prins, 1980, p 96). Lastly, saliva may be used in initiation rituals. Among the Bemba of Zambia, in the initiation ritual (*Cisungu*) young girls perform a ceremony where girls pass ceremonial beads or twigs from girl to girl using their mouths (Richards, 1956, p 72; Maxwell, 1983). These beads and twigs are described as being covered with saliva.

Other researchers have suggested the possibility that the premastication of foods in sub-Saharan African countries may be a behaviour associated with the transfer of saliva from parents to infants and young children. Among the Ovimbundu of Angola, Gladwyn Murray Childs notes that, ‘The mother takes the gruel from the pot and first puts it in her own mouth to cool it with her saliva, then she takes it out and, with the crook of her finger, puts it into the baby's mouth. When she sees that sleep has taken him away, she sucks the gruel from the child's mouth and spits it out on the floor’ (Childs, 1949, p 88). Premastication of foods may occur more commonly using herbs as medicines in the treatment of medical conditions as described above. These herbs may also been taken orally as medicine. It has also been reported among the Banyoro of Uganda that child nurses who were responsible for looking after children often premasticated herbs and then placed them into the mouth of the infant (Roscoe, 1923, p 246). Saliva may also be used to soften the nipples and breasts when feeding an infant as reported among the Igbo of Nigeria (Basden, 1966, p 173).

In designing questionnaires for studies designed to evaluate risk factors for infection with HHV-8 both for children and adults, it is important for investigators to examine culturally specific behavioural practices that involve the exchange of saliva. This review also has relevance for other herpesviruses, which are transmitted via saliva such as Epstein–Barr virus and human herpesviruses 6 and 7. It is likely, as reported here, that practices involving the exchange of saliva will include behaviours associated with healing/treatment of wounds, ritual practices or feeding-related activities. However, as mentioned above, many of these ethnographic studies were conducted decades ago and also are from diverse areas of sub-Saharan Africa and different ethnic groups. Along these lines, it is essential that researchers examine culturally and regionally specific behavioural practices through ethnographic and qualitative means.
